# Socioeconomic and demographic factors influencing dietary patterns in Aktobe: a sample of Kazakhstan’s dietary culture

**DOI:** 10.3389/fpubh.2025.1687222

**Published:** 2025-10-29

**Authors:** Akmaral Baspakova, Elmira Ismagulova, Roza Suleimenova, Gulden Yelgondina, Nadiar M. Mussin, Kulyash R. Zhilisbayeva, Amin Tamadon

**Affiliations:** ^1^Department of Epidemiology, West Kazakhstan Marat Ospanov Medical University, Aktobe, Kazakhstan; ^2^Department of General Hygiene, West Kazakhstan Marat Ospanov Medical University, Aktobe, Kazakhstan; ^3^Department of Public Health and Hygiene, Astana Medical University, Astana, Kazakhstan; ^4^School of General Medicine-2, Asfendiyarov Kazakh National Medical University, Almaty, Kazakhstan; ^5^Department of Surgery No. 2, West Kazakhstan Marat Ospanov Medical University, Aktobe, Kazakhstan; ^6^Department of Languages, West Kazakhstan Marat Ospanov Medical University, Aktobe, Kazakhstan; ^7^Department of Natural Sciences, West Kazakhstan Marat Ospanov Medical University, Aktobe, Kazakhstan

**Keywords:** dietary patterns, socioeconomic factors, demography, Kazakhstan, food habits

## Abstract

**Background:**

Dietary patterns are shaped by a combination of cultural traditions, socioeconomic conditions, and demographic characteristics. In Kazakhstan, rapid economic growth, urbanization, and globalization are influencing food consumption behaviors, with potential implications for public health. Aktobe, the third most populous city in the country, provides a relevant urban context to examine how socioeconomic and demographic factors influence dietary choices within a culturally diverse population.

**Objective:**

Therefore, the aim of this study was to identify the principal dietary patterns among adults in Aktobe, Kazakhstan, using principal component analysis (PCA), and to examine the influence of socioeconomic, demographic, lifestyle, and health-related factors on adherence to these patterns.

**Methods:**

This cross-sectional study was conducted in 2024 among 460 adults aged 18–65 years (210 women, 250 men) recruited from hospitals in Aktobe. Dietary intake was assessed using a culturally adapted food frequency questionnaire, with foods categorized into 11 groups. PCA identified dietary patterns, and negative binomial regression estimated prevalence ratios (PR) for associations between explanatory variables and dietary pattern adherence.

**Results:**

Four dietary patterns were identified: Healthy foods (chicken, fish, green tea, dried fruits, onions), Traditional Kazakh (tea with milk, rice), Bar (processed meats, mayonnaise), and Energy-dense (refined pastries, sweets). Gender, age, and meal frequency were significant predictors. Women showed higher adherence to Healthy foods and Energy-dense patterns, while younger adults adhered less to the Traditional Kazakh pattern but more to the Bar pattern. Shorter intervals between meals and not skipping breakfast were associated with Healthy foods adherence. Oral health, absence of chronic diseases, and better functional status correlated with healthier patterns.

**Conclusion:**

Adults in Aktobe exhibit both traditional and modern dietary patterns, influenced by socioeconomic, demographic, and health factors. Nutrition interventions should be gender- and age-sensitive, preserve beneficial traditional practices, and address the growing consumption of energy-dense processed foods, particularly among younger adults.

## Introduction

Dietary patterns are shaped by a complex interplay of cultural traditions, socioeconomic circumstances, and demographic characteristics (1). Globally, dietary habits are undergoing rapid transformation, influenced by globalization, urbanization, and shifting economic conditions (2). In many countries, this “nutrition transition” is characterized by a gradual shift from traditional diets, often rich in whole foods and culturally specific staples, toward more Westernized dietary habits that include greater amounts of processed, energy-dense foods high in sugar, fat, and salt (3). These changes have important public health implications, as dietary patterns are strongly associated with the risk of chronic diseases such as obesity, cardiovascular disease, type 2 diabetes, and certain cancers (4).

Kazakhstan, the largest landlocked country in the world and a rapidly developing nation in Central Asia, has a rich and diverse food culture shaped by nomadic traditions, regional agricultural practices, and the legacy of the Soviet period (5). Traditional Kazakh diets historically emphasized meat, dairy products, grains, and tea, reflecting the pastoralist lifestyle of much of the population (6). However, in recent decades, Kazakhstan has experienced substantial economic growth, urbanization, and increased exposure to imported food products, leading to notable changes in dietary behavior (7). These shifts have brought both opportunities for diversification and challenges related to the rising consumption of processed and energy-dense foods (8).

Aktobe, located in western Kazakhstan, is the country’s third most populous municipality and an important regional economic hub. Its population includes diverse cultural and ethnic backgrounds, with both urban and peri-urban residents and varying access to traditional and modern food environments. Studying dietary patterns in Aktobe provides an opportunity to examine dietary behaviors in a large, heterogeneous urban center, though findings may not be generalizable to the entire Kazakh population.

Previous studies in other countries have consistently shown that factors such as age, gender, income, education, and household size are significant determinants of dietary choices (9–12). Lifestyle variables—including meal frequency, eating occasions, and food preparation practices—also play a role, as do health-related conditions that may affect food preference and accessibility (1). However, there is limited empirical research on how these factors shape dietary patterns in Kazakhstan, and even less that integrates traditional food culture into the analysis.

Identifying the prevailing dietary patterns in Aktobe and examining their socioeconomic and demographic determinants can provide valuable evidence for public health policy. Understanding who is most likely to adhere to healthier or less healthy dietary patterns can help target nutrition interventions more effectively and support strategies to preserve beneficial traditional dietary practices while addressing emerging nutrition-related health risks.

Therefore, the aim of this study was to identify the principal dietary patterns among adults in Aktobe, Kazakhstan, using principal component analysis (PCA), and to examine the influence of socioeconomic, demographic, lifestyle, and health-related factors on adherence to these patterns. This work contributes to filling a critical gap in the literature on nutrition in Central Asia and provides a foundation for culturally tailored, evidence-based dietary interventions in the region.

## Materials and methods

This study was conducted in Aktobe, Kazakhstan. Data were collected from hospitals across Aktobe in the year 2024. As of May 1, 2024, the population of Aktobe was 942,700. The data collection process involved gathering comprehensive health records and demographic information from the hospitals to ensure a representative sample of the population.

### Study population

This cross-sectional study was conducted in 2024 among adult residents of Aktobe, Kazakhstan, the third most populous municipality in the country. Participants were recruited consecutively from the outpatient departments of major public hospitals across all administrative districts of Aktobe. The sample therefore reflects the urban and peri-urban population of Aktobe, but does not include rural populations outside the city. The sample was therefore non-random (convenience/consecutive sampling) and should be interpreted as representative of the participating hospitals’ outpatient populations rather than the entire city. Eligibility criteria included being aged 18–65 years, a permanent resident of Aktobe, and having the cognitive and physical ability to complete the dietary and health questionnaires. Individuals with acute medical conditions requiring hospitalization at the time of data collection, pregnant or lactating women, and those following medically prescribed diets that substantially altered their habitual intake were excluded.

A total of 460 adults participated in the study, comprising 210 women and 250 men. Informed consent was obtained from all participants prior to data collection. The sample size was determined to provide adequate statistical power for principal component analysis of dietary data and multivariable regression analyses, allowing for stratification by key demographic variables. The study protocol was reviewed and approved by the Ethics Committee of West Kazakhstan Marat Ospanov Medical University. Questionnaires (sociodemographic, lifestyle, health) and the FFQ were completed during the clinic visit via face-to-face interviewer administration using a standardized script.

### Explanatory variables

The independent variables included a range of socioeconomic, demographic, lifestyle, and health-related factors. A brief summary is provided below; detailed operational definitions, measurement methods, disease lists, and category groupings are presented in Appendix A (Variable definitions and coding). All questionnaires were administered face-to-face by trained interviewers in the outpatient departments.

Lifestyle variables captured habitual eating patterns, including number of meals per day (three, four, or five meals), minimum and maximum intervals between meals (hours), days per week without eating breakfast at work (0–5 days), dry food consumption (yes/no), eating just before bedtime (yes/no), and the most satisfying meal of the day (lunch or dinner).

Health-related explanatory variables were derived from clinical measurements, self-reported health assessments, and medical history. These included body mass index (BMI), systolic and diastolic blood pressure, smoking status (number of cigarettes per day), alcohol consumption (mL/week), self-rated health status (excellent, good), and medical visit in the past 3 months (yes/no). Oral health indicators included bleeding gums during cleaning and presence of a large number of carious teeth. Reported symptoms encompassed nausea, loss of appetite, cracks in the corners of the mouth, shortness of breath during exercise, irritability/anxiety/sleep disturbance/memory and attention loss, intolerance to bright light, decreased vision at dusk, dry skin and brittle nails, fatigue/weakness/pain in calf muscles, pain in the right hypochondrium, epigastric pain, and weakening of physical performance.

Chronic disease variables included gastrointestinal diseases, cardiovascular diseases, diseases of the endocrine system, diabetes, liver diseases, kidney diseases, and other chronic diseases. Variables related to healthcare-seeking behavior included consulting with a doctor when unwell (yes/no) and always taking sick leave in case of illness (yes/no).

All explanatory variables were coded and categorized for inclusion in the regression analyses, with reference categories specified for categorical variables. Continuous variables such as BMI, blood pressure, income, and nutritional expenses were used in their measured form or categorized where appropriate for interpretation in the multivariable models.

For regression analysis, categories were collapsed to improve stability: family size (1–2, 3–4, ≥5); minimum and maximum meal intervals (≤3 h, 4–5 h, ≥6 h); days without breakfast at work (0, 1–2, 3–5); and cigarettes/day (non-smoker, 1–4, 5–9, 10–14, ≥15).

### Dietary assessment

Dietary intake was assessed using a structured food frequency questionnaire (FFQ) developed for the Kazakh population and adapted to reflect the local dietary habits of Aktobe residents, administered face-to-face by trained interviewers. This FFQ was culturally adapted and pilot-tested for comprehension in the local population but has not undergone formal validation in this setting; it was administered face-to-face by trained interviewers. The FFQ captured the frequency of consumption of individual food items over the previous month, with response options ranging from “never” to “several times per day.” Portion sizes were standardized using household measures and visual aids to improve accuracy.

The individual food items were subsequently grouped into 11 major food groups—beverages; grains and grain products; legumes; meat and meat products; dairy products; eggs; vegetables; fruits; sweets and desserts; condiments and sauces; and other (sugar)—based on similarity in nutrient profile and culinary use. This classification was used as the basis for identifying dietary patterns through principal component analysis (PCA).

For each participant, the reported frequency of consumption was converted into a standardized measure representing the number of servings per day. The consumption values for all items within each food group were then summed to obtain total daily intake per food group. These food group intakes were standardized (z-scores) prior to PCA to ensure comparability across groups with different measurement scales.

The derived factor scores from PCA represented each participant’s adherence to the identified dietary patterns. For subsequent regression analyses, dietary pattern scores were divided into quartiles, with the 4th quartile representing the highest adherence and the 1st-3rd quartiles combined as the reference category.

### Statistical analysis

Group differences were evaluated using the Student’s t-test (two groups) or ANOVA (≥3 groups) for normally distributed quantitative variables, and the Mann–Whitney U test or Kruskal-Wallis test for non-normally distributed variables. Differences in categorical variables were assessed using the Chi-square test or Fisher’s exact test as appropriate.

Principal Component Analysis (PCA) was employed to identify dietary patterns, followed by varimax orthogonal rotation to simplify the interpretation of extracted factors. The estimation was based on the correlation matrix. The suitability of the dataset for PCA was confirmed by the Kaiser-Meyer-Olkin (KMO) measure of sampling adequacy and the Bartlett test of sphericity (13). Factors were retained based on the following criteria: components with eigenvalues greater than 1.0, the Cattell scree plot, and the conceptual relevance of the identified patterns (11). Each principal component was interpreted based on foods with factor loadings (correlation coefficients between dietary variables and factors) ≥ 0.2 or ≤ − 0.2, which are considered significant contributors to the pattern (12). Negative loadings within a component indicate an inverse association with the food item, while positive loadings indicate a direct association (10). When a food item had loadings ≥0.2 on more than one component, it was assigned to the component with the largest absolute loading, provided the difference between loadings was ≥0.10. Items with ambiguous cross-loadings (<0.10 difference between components) were noted but not used to label or interpret a dietary pattern.

To determine the stability of the factors, the dataset was randomly divided into two subsets, and the same criteria were applied to each subset. The factorial structures of the subgroups were compared to those of the entire sample. The factorial structures of the subgroups were compared to those of the entire sample using Tucker’s congruence coefficient, with values ≥0.90 interpreted as indicating factorial similarity. The principal components were labeled based on the nutritional composition of foods in each factor.

Food group intakes were standardized (z-scores) prior to PCA to ensure comparability across groups. Component (factor) scores were calculated for each participant based on the standardized intakes and the corresponding factor loadings. Technical details of z-standardization and score calculation are provided in Appendix B.

Negative binomial regression with robust variance estimation was used in both bivariate and multivariable analyses to estimate the prevalence ratios (PR) of the independent variables (socioeconomic and demographic factors) in relation to the outcomes, with the dependent variables (dietary patterns) classified as dichotomous: 1st, 2nd, and 3rd quartiles of consumption versus high consumption (4th quartile). Negative binomial regression was preferred over the odds ratio due to the tendency of the latter to overestimate associations when the outcome was not rare (>10%) (14). Variables with *p*-values < 0.20 were included in the multivariable models. To avoid sparse cells and unstable estimates, we recategorized family size, meal intervals, days without breakfast, and cigarettes/day into broader groups as described in Explanatory variables. The detailed bivariate results are provided in Appendix C (Tables C1–C3). Interaction terms between sex and social mobility were tested, and significant models were stratified by sex. Given the high correlation between income and other socioeconomic indicators, models with and without these variables were fitted to test robustness. Quantitative variables were summarized as mean ± standard deviation (SD) when normally distributed or median (interquartile range, IQR) when non-normally distributed, and categorical variables as frequencies and percentages. Group differences were tested using appropriate parametric or non-parametric tests as described below. Estimates of 95% confidence intervals (CI) of the PR were calculated, and the level of significance was set at 0.05. Statistical analyses were performed using the statistical software R version 4.5.0 and RStudio (RStudio 2025.05.0 Build 496).

Due to the large number of predictors and concerns about multicollinearity, sociodemographic/lifestyle variables and health-related variables were modeled in two separate sets of negative binomial regressions. These models are not mutually adjusted for each other, and therefore associations may overlap if predictors are correlated across domains.

## Results

### Classification of food items into dietary groups

Food items were grouped into 11 predefined food groups (beverages; grains; legumes; meat; dairy; eggs; vegetables; fruits; sweets/desserts; condiments/sauces; sugar) as specified in the Methods. The full item-to-group mapping is provided in Table 1.

**Table 1:** Categorization of food items into food groups to study on socioeconomic and demographic factors influencing dietary patterns in Aktobe.

Food group	Items
Beverages	Coffee, Tea with milk, Black tea, Green tea, Milk, Buttermilk, Juices, Carbonated drinks
Grains and grain products	Bread, Buns without cream and filling, Flour products with filling (pies, etc.), Pasta, spaghetti, Dough in hot dishes, Rice, Buckwheat, Semolina
Legumes	Peas, Lentil, Beans
Meat and meat products	Meat and meat products, Fish and fish products, Chicken and chicken products, Sausage and sausage products
Dairy products	Cheese, Cottage cheese, Yoghurt
Eggs	Chicken eggs, Quail eggs
Vegetables	Potato, Carrot, Onion, Cabbage, Beet, Pepper, Courgetti, Eggplants, Cucumbers, Tomato, Broccoli, Lettuce
Fruits	Apples, Bananas, Oranges, Grapes, Kiwi, Strawberry, Raspberry, Cherry, Dried fruits
Sweets and desserts	Pastry products with cream, Pastry products without cream, Cookies, Crackers, Chocolate, Caramel, Marmalade, Jam
Condiments and sauces	Tomato paste, Ketchup, Mayonnaise, Sour cream, Butter
Other	Sugar

### Socioeconomic and demographic characteristics of the study population by gender

Table 2 presents the socioeconomic and demographic characteristics of the study population (*n* = 460). The mean age was 39 years, and most participants were employed and lived in households of three to four members. Average family income was ~170 USD, with ~35% of income spent on food. The overall distribution of meals per day and meal intervals is also shown. Gender-specific distributions are provided in the table for context.

**Table 2:** The socioeconomic and demographic characteristics of adults in Aktobe, categorized by gender.

Factors	Women (*n* = 210)	Men (*n* = 250)	*P*-value	Total
Age (year)	39.2 ± 11.7	39.0 ± 13.4	0.87	-
Age groups (%)			0.35	100
18-24-year-old	12.9	16.4		14.8
25-39-year-old	41.0	37.6		39.1
40-59-year-old	41.0	37.6		39.1
60-65-year-old	5.2	8.4		7.0
Labor status (%)			0.26	100
Works	81.9	85.6		83.9
Does not work	5.7	4.8		5.2
Pensioner	3.8	5.2		4.6
Student	8.6	4.4		6.3
Family size (%)			<0.001	100
1	6.2	14.4		10.7
2	6.7	12.0		9.6
3	38.1	20.0		28.3
4	25.2	24.8		25.0
5	13.3	14.0		13.7
6	8.1	11.6		10.0
7	2.4	3.2		2.8
Monthly family income (USD)	170.2 ± 6.0	173.3 ± 5.5	<0.001	-
Number of meals per day (%)			0.73	100
3	55.7	59.2		57.6
4	39.5	36.0		37.6
5	4.8	4.8		4.8
Minimum interval between meals (hour, %)			0.006	100
2	1.9	8.8		5.7
3	74.3	69.6		71.7
4	23.8	21.6		22.6
Maximum interval between meals (hour, %)			<0.001	100
4	0.0	0.8		0.4
5	9.0	23.6		17.0
6	54.8	63.2		59.3
7	35.2	12.4		22.8
8	1.0	0.0		0.4
Nutritional expenses (USD)	129228.6 ± 33618.1	137100.0 ± 33728.9	0.01	-
Nutritional expenses/total income (%)	34.4 ± 8.2	36.7 ± 9.7	0.008	-
Without eating breakfast at work (days/week, %)			0.005	100
0	24.3	34.0		29.6
1	15.2	8.0		11.3
2	24.8	16.0		20.0
3	8.1	13.6		11.1
4	11.4	10.0		10.7
5	16.2	18.4		17.4
Dry food (%)			0.08	100
Yes	19.4	26.5		23.2
No	80.6	73.5		76.8
Eating just before bedtime (%)			0.55	100
Yes	45.2	48.0		46.7
No	54.8	52.0		53.3
The most satisfying meal (%)			0.61	100
Breakfast	0.0	0.0		0.0
Second breakfast	0.0	0.0		0.0
Lunch	63.3	65.6		64.6
Afternoon snack	0.0	0.0		0.0
Dinner	36.7	34.4		35.4

### Health conditions of the study population by gender

Table 3 summarizes the health conditions of the study population. Mean BMI was 25.6 kg/m^2^, and mean blood pressure values were within the pre-hypertensive range. Most participants rated their health as good or excellent. Selected oral health indicators, symptoms, and chronic disease prevalence are reported in Table 3; gender-specific distributions are provided for completeness.

**Table 3:** The health conditions of adults in Aktobe, categorized by gender.

Factors	Women (*n* = 210)	Men (*n* = 250)	*P*-value	Total
Body mass index (BMI)	25.8 ± 4.2	25.5 ± 4.2	0.11	–
Diastolic blood pressure	77.2 ± 6.4	78.3 ± 5.4	0.05	–
Systolic blood pressure	121.4 ± 7.2	122.1 ± 5.9	0.22	–
Number of cigarettes smoked per day (%)			0.34	100
0	79.4	62.4		41.9
5	2.4	1.9		4.3
6	6.2	9.5		15.7
7	4.8	2.9		7.6
8	1.0	4.8		5.7
10	2.9	11.4		14.3
15	3.3	7.1		10.5
Alcohol consumption (mL/week)	775.0 ± 351.2 (*n* = 16)	3095.5 ± 5611.9 (*n* = 44)	0.11	–
Health assessment categories (%)			0.77	100
Excellent	51.4	52.8		52.2
Good	48.6	47.2		47.8
Bad	0.0	0.0		0.0
Do not know	0.0	0.0		0.0
Visiting doctor in the past 3 months (%)			0.08	100
Yes	75.2	82.0		78.9
No	24.8	18.0		21.1
Bleeding gums during cleaning (%)			0.48	100
No	50.0	54.4		52.4
Sometimes	44.3	38.8		41.3
Often	5.7	6.8		6.3
A large number of carious teeth (%)			0.02	100
No	61.9	54.0		57.6
Sometimes	36.2	38.8		37.6
Often	1.9	7.2		4.8
Nausea (%)			0.07	100
No	90.5	94.8		92.8
Sometimes	9.5	5.2		7.2
Often	0.0	0.0		0.0
Loss of appetite (%)			0.32	100
No	87.6	84.4		85.9
Sometimes	12.4	15.6		14.1
Often	0.0	0.0		0.0
Cracks in the corners of the mouth (%)			0.55	100
No	69.0	66.4		67.6
Sometimes	31.0	33.6		32.4
Often	0.0	0.0		0.0
Shortness of breath during exercise (%)			0.47	100
No	44.3	38.8		41.3
Sometimes	42.4	45.6		44.1
Often	13.3	15.6		14.6
Irritability, anxiety, sleep disturbance, memory, and attention loss (%)			0.04	100
No	35.7	47.2		42.0
Sometimes	49.0	41.6		45.0
Often	15.2	11.2		13.0
Intolerance to bright light (%)			0.11	100
No	81.4	75.2		78.0
Sometimes	18.6	24.8		22.0
Often	0.0	0.0		0.0
Decreased vision at dusk (%)			0.12	100
No	58.1	48.4		52.8
Sometimes	19.1	26.4		23.1
Often	22.9	25.2		24.1
Dry skin, brittle nails (%)			0.05	100
No	77.6	83.6		80.9
Sometimes	10.5	10.8		10.7
Often	11.9	5.6		8.5
Fatigue, weakness, pain in the calf muscles (%)			0.01	100
No	69.5	80.8		75.7
Sometimes	20.5	14.8		17.4
Often	10.0	4.4		7.0
Pain in the right hypochondrium (%)			0.79	100
No	91.9	91.2		91.5
Sometimes	8.1	8.8		8.5
Often	0.0	0.0		0.0
Epigastric pain (%)			0.03	100
No	82.4	90.8		87.0
Sometimes	11.0	6.0		8.3
Often	6.7	3.2		4.8
Weakening of physical performance (%)			0.003	100
No	15.7	26.4		21.5
Sometimes	77.1	62.4		69.1
Often	7.1	11.2		9.3
Mental disability (%)			0.04	100
No	51.4	56.8		54.3
Sometimes	47.6	39.2		43.0
Often	1.0	4.0		2.6
The frequency of colds during the year (%)			0.05	100
No more than 1 time	13.8	20.8		17.6
2 or more times	86.2	79.2		82.4
Consult with a doctor if unwell (%)			0.88	100
Yes	64.3	63.6		63.9
No	35.7	36.4		36.1
Always take sick leave in case of illness (%)			0.12	100
Yes	85.2	79.6		82.2
No	14.8	20.4		17.8
Gastrointestinal diseases (%)			0.04	100
Yes	44.3	54.0		49.6
No	55.7	46.0		50.4
Cardiovascular diseases (%)			0.50	100
Yes	84.8	82.4		83.5
No	15.2	17.6		16.5
Diseases of the endocrine system (%)			0.001	100
Yes	85.2	94.4		90.2
No	14.8	5.6		9.8
Kidney diseases (%)			ND	100
Yes	0.0	0.0		0.0
No	100	100		100
Diabetes (%)			0.27	100
Yes	98.1	96.4		97.2
No	1.9	3.6		2.8
Liver diseases (%)			0.003	100
Yes	100	96.0		97.8
No	0	4.0		2.2
Other diseases (%)			0.07	100
Yes	83.8	89.6		87.0
No	16.2	10.4		13.0

### Principal dietary patterns identified in adults from Aktobe

Principal component analysis identified four distinct dietary patterns (Table 4). The Healthy foods pattern was characterized by poultry, fish, green tea, and dried fruits. The Traditional Kazakh pattern reflected tea with milk and rice, alongside lower intake of some dairy and grains. The Bar pattern included processed meats and mayonnaise. The Energy-dense pattern was defined by pastries and sweets. Together, these four patterns accounted for 16.4% of the variance in dietary intake.

**Table 4:** Distribution of factor loadings for principal dietary patterns identified among adults in Aktobe.

Food groups	Healthy foods	Traditional Kazakh	Bar	Energy-dense
Chicken and chicken products	0.530			
Green tea	0.507			
Dried fruits	0.382			
Onion	0.231			
Fish and fish products	0.229			
Eggplants	−0.224			
Courgetti	−0.230			
Cucumbers	−0.245			
Beans	−0.248			
Cabbage	−0.331			
Carrot	−0.344			
Rice		0.200		
Tea with milk		0.283		
Cottage cheese		−0.259		
buttermilk		−0.272		
Buckwheat		−0.288		
Chicken eggs		−0.374		
mayonnaise			0.501	
Sausage and sausage products			0.380	
Carbonated drinks			−0.248	
Black tea			−0.465	
Coffee			−0.538	
Pastry products without cream				−0.209
Jam				−0.240
Caramel				−0.259
Buns without cream and filling				−0.292
Flour products with filling (pies, etc.)				−0.385
Explained variance (%)	9.09	20.0	20.0	16.67
Eigenvalue	11.0	5.0	5.0	6.0

Only foods with factor loadings ≥ 0.2 or ≤ − 0.2 were shown. Total explained variance = 16.44%.

### Socioeconomic and demographic factors associated with dietary patterns

Results are presented separately for sociodemographic/lifestyle factors (Table 5) and health-related factors (Table 6). These models should be interpreted independently, as they were not adjusted for predictors from the other domain, and some associations may intersect.

**Table 5:** Prevalence ratios (PR*) and confidence intervals (95% CI) of socioeconomic and demographic factors influencing dietary patterns in Aktobe.

Factors	Healthy foods	Traditional Kazakh	Bar	Energy-dense
Sex				
Female	Reference	Reference	Reference	Reference
Male	0.89 (0.74–1.06) *p* = 0.15	1.12 (0.96–1.28) *p* = 0.19	0.78 (0.66–0.92) *p* = 0.003	0.77 (0.65–0.91) *p* = 0.003
Age groups (%)				
18-24-year-old	0.85 (0.56–1.30); *p* = 0.450	0.68 (0.50–0.92); *p* = 0.020	1.49 (0.99–2.23); *p* = 0.060	0.90 (0.60–1.34); *p* = 0.580
25-39-year-old	1.01 (0.70–1.46); *p* = 0.950	0.76 (0.58–0.99); *p* = 0.040	1.77 (1.22–2.56); *p* = 0.002	1.12 (0.78–1.58); *p* = 0.550
40-59-year-old	0.97 (0.67–1.40); *p* = 0.880	0.69 (0.52–0.90); *p* = 0.007	1.60 (1.11–2.32); *p* = 0.010	1.06 (0.74–1.51); *p* = 0.760
60-65-year-old	Reference	Reference	Reference	Reference
Labor status (%)				
Works	1.27 (0.84–1.92); *p* = 0.25	1.08 (0.70–1.67); *p* = 0.73	0.77 (0.51–1.17); *p* = 0.23	1.13 (0.73–1.75); *p* = 0.57
Does not work	1.22 (0.68–2.19); *p* = 0.51	1.13 (0.61–2.07); *p* = 0.70	0.74 (0.40–1.36); *p* = 0.33	1.08 (0.58–2.00); *p* = 0.80
Pensioner	1.32 (0.73–2.41); *p* = 0.36	1.25 (0.67–2.36); *p* = 0.50	0.54 (0.28–1.03); *p* = 0.06	0.69 (0.36–1.48); *p* = 0.27
Student	Reference	Reference	Reference	Reference
Family size (%)				
1–2	0.73 (0.38–1.23); *p* = 0.35	1.92 (0.91–4.05); *p* = 0.09	0.64 (0.32–1.27); *p* = 0.20	1.05 (0.70–2.12); *p* = 0.40
3–4	0.86 (0.47–1.57); *p* = 0.65	2.00 (1.00–4.01); *p* = 0.05	0.95 (0.52–1.73); *p* = 0.85	1.20 (0.64–2.23); *p* = 0.60
≥5	0.90 (0.47–1.70); *p* = 0.74	1.84 (0.82–3.82); *p* = 0.11	0.64 (0.32–1.27); *p* = 0.22	1.16 (0.61–2.22); *p* = 0.65
7	Reference	Reference	Reference	Reference
Number of meals per day (%)				
3	1.12 (0.70–1.77); *p* = 0.66	1.51 (0.89–2.56); *p* = 0.13	1.06 (0.64–1.77); *p* = 0.81	1.59 (0.93–2.66); p = 0.09
4	1.19 (0.73–1.91); *p* = 0.48	1.80 (1.05–3.07); *p* = 0.03	1.43 (0.86–2.41); *p* = 0.17	1.80 (1.06–3.29); *p* = 0.03
5	Reference	Reference	Reference	Reference
Minimum interval between meals (hour, %)				
2	1.71 (1.08–2.72); p = 0.02	1.48 (0.91–2.38); p = 0.11	0.91 (0.56–1.51); p = 0.74	0.63 (0.38–1.04); *p* = 0.07
3	1.73 (1.36–2.20); *p* < 0.001	1.09 (0.84–1.40); *p* = 0.50	1.02 (0.79–1.32); *p* = 0.85	0.95 (0.74–1.22); *p* = 0.69
4	Reference	Reference	Reference	Reference
Maximum interval between meals (hour, %)				
≤3 h	0.37 (0.08–1.65); *p* = 0.20	0.90 (0.11–9.03); *p* = 0.91	0.30 (0.03–2.72); *p* = 0.28	1.00 (0.06–16.44); *p* = 1.00
4–5 h	0.47 (0.11–2.01); *p* = 0.30	0.39 (0.08–1.82); *p* = 0.22	0.33 (0.07–1.49); *p* = 0.18	3.00 (0.45–22.20); *p* = 0.25
≥6 h	Reference	Reference	Reference	Reference
Without eating breakfast at work (days/week, %)				
0 days	1.51 (1.13–2.03); *p* = 0.007	1.26 (0.92–1.72); *p* = 0.15	0.94 (0.68–1.28); *p* = 0.70	0.90 (0.65–1.23); *p* = 0.52
1–2 days	1.17 (0.90–1.52); *p* = 0.20	0.90 (0.64–1.28); *p* = 0.45	1.07 (0.78–1.49); *p* = 0.65	1.05 (0.78–1.42); *p* = 0.70
3–5 days	Reference	Reference	Reference	Reference
Dry food (%)				
No	1.34 (1.06–1.70); *p* = 0.02	1.00 (0.78–1.28); *p* = 0.99	1.14 (0.89–1.48); *p* = 0.30	0.94 (0.73–1.21); *p* = 0.66
Yes	Reference	Reference	Reference	Reference
Eating just before bedtime (%)				
No	0.93 (0.77–1.14); *p* = 0.50	1.15 (0.93–1.42); *p* = 0.19	1.01 (0.82–1.25); *p* = 0.92	1.04 (0.85–1.28); *p* = 0.68
Yes	Reference	Reference	Reference	Reference
The most satisfying meal (%)				
Breakfast	ND	ND	ND	ND
Second breakfast	ND	ND	ND	ND
Lunch	1.03 (0.84–1.26); *p* = 0.81	0.93 (0.75–1.16); *p* = 0.53	1.06 (0.85–1.31); *p* = 0.62	0.92 (0.75–1.15); *p* = 0.47
Afternoon snack	ND	ND	ND	ND
Dinner	Reference	Reference	Reference	Reference

ND, no data; category absent or too few cases to allow estimation. * Values are prevalence ratios (PR) with 95% confidence intervals. PRs were obtained by exponentiating coefficients from log-link generalized linear models with robust variance; 95% CIs were exponentiated from the coefficient confidence limits. Reference categories are PR = 1.00.

**Table 6:** Prevalence ratios (PR*) and confidence intervals (95% CI) of the health conditions influencing dietary patterns in Aktobe.

Factors	Healthy foods	Traditional Kazakh	Bar	Energy-dense
Number of cigarettes smoked per day (%)				
0	1.01 (0.64–1.62); *p* = 0.95	1.49 (0.89–2.49); *p* = 0.13	0.63 (0.39–1.01); *p* = 0.05	1.39 (0.84–2.29); *p* = 0.19
1–9	0.78 (0.45–1.35); *p* = 0.35	1.42 (0.82–2.46); *p* = 0.22	0.78 (0.41–1.49); *p* = 0.40	0.90 (0.50–2.01); *p* = 0.75
10–15	1.08 (0.61–1.96); *p* = 0.78	1.63 (0.85–3.10); *p* = 0.14	0.53 (0.29–1.00); *p* = 0.05	0.75 (0.39–1.45); *p* = 0.40
>15	Reference	Reference	Reference	Reference
Health assessment categories (%)				
Excellent	0.91 (0.75–1.10); *p* = 0.35	1.07 (0.87–1.32); *p* = 0.51	1.06 (0.86–1.31); *p* = 0.57	0.90 (0.73–1.10); *p* = 0.30
Good	ND	ND	ND	ND
Bad	ND	ND	ND	ND
Do not know	Reference	Reference	Reference	Reference
Visiting doctor in the past 3 months (%)				
Yes	Reference	Reference	Reference	Reference
No	0.90 (0.71–1.15); *p* = 0.42	1.19 (0.91–1.52); *p* = 0.21	1.04 (0.81–1.35); *p* = 0.74	0.98 (0.75–1.26); *p* = 0.86
Bleeding gums during cleaning (%)				
No	1.52 (1.01–2.32); *p* = 0.05	0.73 (0.47–1.12); *p* = 0.14	1.57 (0.98–2.48); *p* = 0.06	1.00 (0.64–1.55); *p* = 0.99
Sometimes	1.36 (0.89–2.08); *p* = 0.16	0.76 (0.46–1.19); *p* = 0.23	1.58 (0.99–2.53); *p* = 0.06	1.27 (0.82–1.99); *p* = 0.28
Often	Reference	Reference	Reference	Reference
A large number of carious teeth (%)				
No	2.06 (1.27–3.35); *p* = 0.003	1.26 (0.76–2.10); *p* = 0.37	1.96 (1.12–3.36); *p* = 0.02	1.03 (0.63–1.69); *p* = 0.91
Sometimes	1.58 (0.96–2.59); *p* = 0.07	1.32 (0.79–2.21); *p* = 0.30	1.86 (1.07–3.23); *p* = 0.03	0.93 (0.57–1.54); *p* = 0.78
Often	Reference	Reference	Reference	Reference
Nausea (%)				
No	1.11 (0.76–1.62); *p* = 0.61	0.99 (0.66–1.49); *p* = 0.97	0.55 (0.38–0.81); *p* = 0.003	1.45 (0.95–2.20); *p* = 0.08
Sometimes	ND	ND	ND	ND
Often	Reference	Reference	Reference	Reference
Loss of appetite (%)				
No	0.80 (0.61–1.06); *p* = 0.12	1.09 (0.81–1.48); *p* = 0.54	1.00 (0.74–1.35); *p* = 0.99	1.23 (0.91–1.66); *p* = 0.18
Sometimes	ND	ND	ND	ND
Often	Reference	Reference	Reference	Reference
Cracks in the corners of the mouth (%)				
No	0.84 (0.68–1.04); *p* = 0.11	0.91 (0.73–1.14); *p* = 0.43	0.99 (0.79–1.24); *p* = 0.97	0.98 (0.79–1.22); *p* = 0.83
Sometimes	ND	ND	ND	ND
Often	Reference	Reference	Reference	Reference
Shortness of breath during exercise (%)				
No	1.39 (1.03–1.88); *p* = 0.03	0.89 (0.64–1.21); *p* = 0.44	1.60 (1.15–2.23); *p* = 0.005	1.05 (0.76–1.45); *p* = 0.75
Sometimes	1.26 (0.93–1.70); *p* = 0.13	1.02 (0.75–1.40); *p* = 0.89	1.48 (1.06–2.06); *p* = 0.02	1.35 (0.98–1.84); *p* = 0.07
Often	Reference	Reference	Reference	Reference
Irritability, anxiety, sleep disturbance, memory, and attention loss (%)				
No	1.01 (0.74–1.38); *p* = 0.93	0.87 (0.63–1.21); *p* = 0.41	0.84 (0.60–1.17); *p* = 0.31	1.08 (0.78–1.50); *p* = 0.63
Sometimes	0.89 (0.65–1.21); *p* = 0.44	0.76 (0.55–1.04); *p* = 0.09	1.21 (0.87–1.68); *p* = 0.25	1.21 (0.85–1.66); *p* = 0.27
Often	Reference	Reference	Reference	Reference
Intolerance to bright light (%)				
No	0.90 (0.71–1.14); *p* = 0.36	1.09 (0.84–1.40); *p* = 0.50	0.91 (0.70–1.17); *p* = 0.47	1.14 (0.89–1.46); *p* = 0.31
Sometimes	ND	ND	ND	ND
Often	Reference	Reference	Reference	Reference
Decreased vision at dusk (%)				
No	1.12 (0.88–1.42); *p* = 0.38	1.07 (0.83–1.39); *p* = 0.57	1.51 (1.16–1.95); *p* = 0.002	0.80 (0.62–1.09); *p* = 0.09
Sometimes	1.19 (0.90–1.57); *p* = 0.24	1.12 (0.83–1.51); *p* = 0.48	1.43 (1.05–1.95); *p* = 0.02	0.73 (0.54–0.99); *p* = 0.04
Often	Reference	Reference	Reference	Reference
Dry skin, brittle nails (%)				
No	0.99 (0.70–1.41); *p* = 0.96	1.06 (0.73–1.54); *p* = 0.77	0.90 (0.62–1.32); *p* = 0.62	0.66 (0.46–0.94); *p* = 0.02
Sometimes	1.12 (0.72–1.74); *p* = 0.62	1.22 (0.76–1.97); *p* = 0.40	1.12 (0.69–1.80); *p* = 0.65	0.56 (0.35–0.91); *p* = 0.02
Often	Reference	Reference	Reference	Reference
Fatigue, weakness, pain in the calf muscles (%)				
No	0.95 (0.64–1.40); *p* = 0.80	0.95 (0.63–1.43); *p* = 0.80	1.31 (0.85–2.01); *p* = 0.22	0.67 (0.45–1.00); *p* = 0.05
Sometimes	1.05 (0.68–1.63); *p* = 0.82	0.82 (0.52–1.31); *p* = 0.41	1.91 (1.19–3.10); *p* = 0.008	0.80 (0.51–1.26); *p* = 0.35
Often	Reference	Reference	Reference	Reference
Pain in the right hypochondrium (%)				
No	1.09 (0.77–1.55); *p* = 0.62	0.90 (0.62–1.31); *p* = 0.58	1.23 (0.84–1.80); *p* = 0.29	0.91 (0.63–1.32); *p* = 0.64
Sometimes	ND	ND	ND	ND
Often	Reference	Reference	Reference	Reference
Epigastric pain (%)				
No	0.99 (0.62–1.55); *p* = 0.96	1.30 (0.79–2.14); *p* = 0.31	1.35 (0.81–2.25); *p* = 0.25	0.58 (0.37–0.93); *p* = 0.02
Sometimes	0.99 (0.57–1.74); *p* = 0.98	1.16 (0.63–2.14); *p* = 0.64	1.75 (0.95–3.23); *p* = 0.07	0.84 (0.48–1.49); *p* = 0.57
Often	Reference	Reference	Reference	Reference
Weakening of physical performance (%)				
No	2.69 (1.81–4.01); *p* < 0.001	1.36 (0.90–2.08); *p* = 0.14	1.31 (0.85–2.01); *p* = 0.22	0.83 (0.55–1.23); *p* = 0.35
Sometimes	2.08 (1.46–2.97); *p* < 0.001	1.34 (0.91–1.94); *p* = 0.14	1.68 (1.15–2.48); *p* = 0.008	1.04 (0.73–1.49); *p* = 0.82
Often	Reference	Reference	Reference	Reference
Mental disability (%)				
No	2.83 (1.45–5.54); *p* = 0.002	1.75 (0.86–3.59); *p* = 0.12	2.48 (1.16–5.31); *p* = 0.02	0.94 (0.49–1.80); *p* = 0.86
Sometimes	2.21 (1.13–4.32); *p* = 0.022	1.84 (0.90–3.79); *p* = 0.09	2.44 (1.13–5.21); *p* = 0.02	0.91 (0.47–1.75); *p* = 0.78
Often	Reference	Reference	Reference	Reference
The frequency of colds during the year (%)				
No more than 1 time	0.83 (0.64–1.07); *p* = 0.15	1.02 (0.77–1.34); *p* = 0.91	0.98 (0.74–1.28); *p* = 0.86	0.83 (0.62–1.09); *p* = 0.17
2 or more times	Reference	Reference	Reference	Reference
Consult with a doctor if unwell (%)				
No	1.01 (0.83–1.23); *p* = 0.92	1.36 (1.09–1.71); *p* = 0.006	0.97 (0.79–1.22); *p* = 0.82	1.16 (0.94–1.43); *p* = 0.18
Yes	Reference	Reference	Reference	Reference
Always take sick leave in case of illness (%)				
No	0.96 (0.74–1.26); *p* = 0.74	0.83 (0.63–1.08); *p* = 0.17	1.00 (0.78–1.31); *p* = 0.98	1.46 (1.11–1.92); *p* = 0.008
Yes	Reference	Reference	Reference	Reference
Gastrointestinal diseases (%)				
No	0.86 (0.71–1.04); *p* = 0.13	0.94 (0.76–1.16); *p* = 0.55	0.91 (0.74–1.13); *p* = 0.42	0.82 (0.67–1.01); *p* = 0.06
Yes	Reference	Reference	Reference	Reference
Cardiovascular diseases (%)				
No	0.92 (0.71–1.20); *p* = 0.55	0.91 (0.69–1.21); *p* = 0.54	1.45 (1.08–1.94); *p* = 0.01	1.68 (1.26–2.25); *p* = 0.001
Yes	Reference	Reference	Reference	Reference
Diseases of the endocrine system (%)				
No	1.73 (1.24–2.44); *p* = 0.002	1.23 (0.87–1.77); *p* = 0.24	0.76 (0.54–1.08); *p* = 0.13	0.79 (0.56–1.10); *p* = 0.17
Yes	Reference	Reference	Reference	Reference
Kidney diseases (%)				
No	ND	ND	ND	ND
Yes	Reference	Reference	Reference	Reference
Diabetes (%)				
No	1.27 (0.70–2.32); *p* = 0.42	0.67 (0.37–1.23); *p* = 0.20	0.71 (0.38–1.29); *p* = 0.26	0.99 (0.53–1.84); *p* = 0.97
Yes	Reference	Reference	Reference	Reference
Liver diseases (%)				
No	0.69 (0.36–1.34); *p* = 0.27	0.52 (0.26–1.02); *p* = 0.06	1.30 (0.62–2.72); *p* = 0.49	1.26 (0.61–2.61); *p* = 0.53
Yes	Reference	Reference	Reference	Reference
Other diseases (%)				
No	1.03 (0.77–1.41); *p* = 0.83	0.91 (0.68–1.25); *p* = 0.58	0.88 (0.64–1.19); *p* = 0.40	0.87 (0.64–1.17); *p* = 0.36
Yes	Reference	Reference	Reference	Reference

ND, no data; category absent or too few cases to allow estimation. * Values are prevalence ratios (PR) with 95% confidence intervals. PRs were obtained by exponentiating coefficients from log-link generalized linear models with robust variance; 95% CIs were exponentiated from the coefficient confidence limits. Reference categories are PR = 1.00.

Detailed bivariate regression results for all predictors are presented in Appendix C (Tables C1–C3). Analyses used collapsed categories for family size, meal intervals, days without breakfast, and cigarettes/day (see Methods). Full estimates are in Table 5. Only variables with bivariate *p* < 0.20 were considered in multivariable models reported in the main text. Negative binomial regression analysis was performed after Poisson regression diagnostics indicated overdispersion. The model estimated prevalence ratios (PR) and 95% confidence intervals (CI) for associations between socioeconomic and demographic characteristics and the four identified dietary patterns. The detailed results are presented in Table 5. Some categories could not be estimated due to absence of observations; these are shown as ND in Table 5. Findings are summarized by dietary pattern: Healthy foods, Traditional Kazakh, Bar, Energy-dense (Table 5).

#### Healthy foods

Being male was not significantly associated with intake (PR = 0.89; 95% CI: 0.74–1.06; *p* = 0.15). Shorter minimum intervals (≤3 h) and not skipping breakfast (0 days/week) were associated with higher adherence (Table 5).

#### Traditional Kazakh

Compared with adults aged 60–65 years, younger groups (18–24, 25–39, 40–59) showed lower adherence. Consuming four meals/day was positively associated (Table 5).

#### Bar

Men had lower adherence than women. Adults aged 25–39 and 40–59 years showed higher adherence than those aged 60–65 years (Table 5).

#### Energy-dense

Men had lower adherence than women. Consuming four meals/day and skipping breakfast more days/week (3–5 vs. 0) were associated with adherence (Table 5). Overall, the analysis highlighted that meal frequency, meal timing, and certain lifestyle habits were significant predictors of dietary pattern adherence, while gender and age influenced adherence to specific patterns.

### Health conditions associated with dietary patterns

Negative binomial regression analysis assessed the relationship between health conditions and adherence to the four identified dietary patterns. The full results are shown in Table 6. Some categories could not be estimated due to absence of observations; these are shown as ND in Table 6. Findings are summarized by dietary pattern: Healthy foods, Traditional Kazakh, Bar, Energy-dense (Table 6).

#### Healthy foods

Lower adherence was observed in smokers (5/day). Better oral health (no bleeding gums; fewer carious teeth), no shortness of breath, and absence of endocrine disease were positively associated (Table 6).

#### Traditional Kazakh

No strong positive associations; borderline findings for 7/day smoking and absence of mental disability (Table 6).

#### Bar

Negative associations for 10/day smoking and nausea; positive associations for better oral health and absence of cardiovascular disease (Table 6).

#### Energy-dense

Negative associations with absence of dermatologic symptoms and fatigue; positive association for not always taking sick leave and absence of cardiovascular disease (Table 6).

Overall, the analysis indicated that oral health, absence of chronic conditions, and certain symptom profiles were linked to healthier dietary patterns, while symptoms such as nausea, fatigue, and dermatological problems were inversely associated with adherence to healthier or energy-dense dietary patterns.

## Discussion

This study identified four distinct dietary patterns among adults in Aktobe—Healthy foods, Traditional Kazakh, Bar, and Energy-dense—and explored their associations with socioeconomic, demographic, lifestyle, and health-related factors. The findings provide new insights into the dietary behaviors of a Kazakh urban population, highlighting the influence of both traditional cultural practices and modern dietary transitions on food choices.

The Healthy foods pattern was characterized by higher consumption of chicken, fish, green tea, dried fruits, and vegetables such as onions, and lower intake of starchy vegetables and legumes. This pattern aligns with globally recognized healthy eating patterns that emphasize lean proteins, antioxidant-rich beverages, and nutrient-dense plant-based foods (15). The Traditional Kazakh pattern reflected culturally specific elements, notably tea with milk and rice, combined with a lower consumption of certain dairy products, buckwheat, and eggs. This likely reflects the historical pastoralist dietary heritage of Kazakhstan, in which tea with milk remains a daily staple but regional grain and dairy preferences vary. The Bar pattern, marked by processed meats and mayonnaise and lower intake of coffee and black tea, suggests social eating occasions and higher exposure to processed, savory foods. The Energy-dense pattern consisted primarily of refined carbohydrate-based snacks and sweets, consistent with Westernized snacking habits that have become more prevalent in urban Central Asia (16). The coexistence of traditional and modern dietary patterns in Aktobe reflects broader nutrition transition processes observed in post-Soviet contexts, where rapid economic changes, increased food imports, and lifestyle shifts contribute to diverse but sometimes conflicting dietary influences.

Our findings demonstrate that gender, age, and meal frequency were significant determinants of dietary pattern adherence. Men had lower adherence to the Healthy foods and Energy-dense patterns, which may reflect gendered food preferences and health awareness differences, as observed in other Central Asian and Eastern European populations (17). Women tended to report more frequent consumption of nutrient-dense foods, consistent with literature showing that women are generally more health-conscious and responsive to dietary guidelines (18).

Age differences were most evident in the Traditional Kazakh and Bar patterns. Younger adults (18–39 years) were less likely to follow the Traditional Kazakh pattern but more likely to adhere to the Bar pattern, indicating a generational shift toward more social, convenience-oriented eating behaviors and away from traditional meal structures. This generational dietary divergence has been reported in other transitional economies, where urban youth adopt globalized food practices more rapidly than older adults (19).

Meal frequency and timing also emerged as important predictors. Consuming four meals per day was positively associated with both the Traditional Kazakh and Energy-dense patterns, suggesting that an increased number of eating occasions may facilitate both traditional multi-course meals and high-calorie snack consumption. Shorter intervals between meals were associated with greater adherence to the Healthy foods pattern, potentially indicating more structured eating schedules among those prioritizing healthful foods (20).

It is important to note that some predictors identified in our regression models, such as meal frequency and breakfast skipping, may not act as independent causal factors influencing dietary pattern adherence, but instead may be integral elements of the same dietary culture. For example, the Energy-dense pattern, characterized by refined pastries and sweets, may naturally coincide with more frequent eating occasions or snacking habits. Therefore, these associations should be interpreted as reflecting correlated behaviors within broader food cultures, rather than as causal determinants of the dietary patterns.

Oral health indicators and chronic disease status were strongly associated with dietary patterns. The absence of carious teeth and bleeding gums was linked to greater adherence to the Healthy foods and bar patterns, suggesting a potential bidirectional relationship between diet quality and oral health. While healthier diets support better oral status, individuals with better oral health may also be more able to consume a variety of foods, including those requiring more chewing, such as fresh fruits and vegetables (21).

The Healthy foods pattern was also positively associated with the absence of endocrine disorders and fewer functional limitations, supporting evidence that nutrient-rich diets contribute to better metabolic and physical health (22). Conversely, symptoms such as fatigue, dermatological problems, and gastrointestinal complaints were inversely associated with the Energy-dense pattern, reinforcing the link between high-sugar, refined-carbohydrate diets and poorer overall health status (23).

The Bar pattern showed a complex relationship with health variables. While certain health conditions were positively associated, the pattern’s processed meat and mayonnaise content may carry long-term health risks, as reported in studies linking such foods to increased cardiometabolic risk (24).

Comparable studies in neighboring countries provide useful context for interpreting our results. In Russia, PCA-based analyses have identified dietary patterns labelled “Meat,” “Mixed,” or “Rational,” which include traditional staples such as potatoes, bread, and tea alongside increasing prominence of processed meats and sweets, similar to our “Traditional Kazakh” and “Energy-dense” patterns (25). Studies in Mongolia (26) and Uzbekistan (27) are fewer but suggest diets with heavy reliance on bread, meat, and dairy products, reflecting cultural legacies from Soviet and nomadic food systems. In Iran, analyses over time also reveal coexisting traditional diets rich in rice, bread, and tea as well as “Western” patterns characterized by fast foods, sugary snacks, and processed products. For example, Aghayan et al. (28) found that between 2006 and 2017, Western dietary patterns increased in Iranian adults while traditional/habitual patterns persisted. Overall, the coexistence of traditional staples with energy-dense modern foods appears common across Central Asia and nearby Asian regions, suggesting that the nutrition transition seen in Aktobe reflects broader regional dynamics. Nevertheless, unique cultural elements, such as the prominence of tea with milk in Kazakh diets, mark local distinctions.

These findings have important implications for public health strategies in Kazakhstan. First, nutrition interventions should be gender-sensitive, as women were more likely to follow healthier diets, while men emerged as a priority group for dietary improvement campaigns—particularly those aimed at reducing processed food consumption and increasing the intake of nutrient-dense foods. Second, although adherence to the Traditional Kazakh pattern has declined among younger adults, preserving its healthier elements, such as tea with milk and structured meal patterns, and adapting them to meet modern nutritional recommendations could help maintain cultural dietary heritage while promoting health. Third, given the observed associations between oral health indicators and dietary patterns, integrating oral health initiatives with nutrition programs may provide synergistic benefits for overall well-being. Finally, the growing popularity of energy-dense, Western-style snacking patterns underscores the need for policies that encourage the consumption of whole foods and restrict the marketing of high-sugar, high-fat processed snacks, particularly to younger populations who are most susceptible to these dietary trends.

The study’s strengths include the use of PCA to derive culturally relevant dietary patterns, the inclusion of a broad set of socioeconomic and health-related explanatory variables, and the application of negative binomial regression to address overdispersion in the data. The urban sample from Aktobe provides valuable insights into dietary behaviors in a rapidly developing Kazakh city.

Future studies should extend this analysis to rural populations and other regions of Kazakhstan to capture national dietary diversity. Longitudinal designs could clarify causal relationships between socioeconomic changes, dietary patterns, and health outcomes. Intervention studies testing targeted dietary education programs—particularly for men and younger adults—would be valuable. Finally, incorporating objective dietary biomarkers could validate self-reported dietary intake and strengthen the evidence base for nutrition policy.

### Limitations

This study has several limitations that should be considered when interpreting the findings. First, the cross-sectional design precludes causal inference, and dietary intake was assessed by self-reported FFQs, which are subject to recall bias and do not account for seasonal variation in food availability. Second, the sample was recruited from outpatient hospital departments in Aktobe, using non-random sampling, which may limit representativeness; individuals not seeking medical care or those living in rural areas were not included. Third, while PCA is a useful tool to identify prevailing dietary patterns, it explains only part of the variance in food intake and cannot capture all individual-level complexity. Fourth, because the study was conducted in a single large urban center, findings cannot be generalized to the entire Kazakh population. Kazakhstan is geographically vast and culturally diverse, with regional differences in socioeconomic conditions, ethnic composition, and food culture that may lead to different dietary patterns elsewhere. In addition, we did not collect data on ethnicity or education level, both of which are important determinants of diet, and the FFQ, although adapted and pilot-tested for local use, has not undergone formal validation in this population. Taken together, these factors suggest that our results should be interpreted with caution, and future multi-regional and longitudinal studies are needed to confirm these findings and explore dietary diversity across Kazakhstan. Because recruitment was limited to hospitals in Aktobe, our findings represent an urban hospital-based population. They cannot be generalized to rural areas of Kazakhstan, where food environments and cultural dietary practices may differ substantially.

Because sociodemographic/lifestyle and health-related predictors were modeled separately to reduce dimensionality and avoid unstable estimates, the associations obtained should be interpreted as descriptive rather than independent effects. Some associations may be explained by correlated predictors across models. Future studies with larger multi-regional samples could use integrated modeling strategies, including stepwise regression or dimension reduction techniques, to account for these overlaps.

In addition, while the FFQ used in this study was culturally adapted and pilot-tested for local comprehension, it has not undergone formal validation in this population. This may introduce measurement error in reported intakes and should be considered when interpreting our findings.

## Conclusion

In summary, this study identified four distinct dietary patterns in Aktobe, reflecting a mix of traditional Kazakh and modern dietary influences. Socioeconomic and demographic factors, especially gender, age, meal frequency, and health status, significantly shaped adherence to these patterns. Public health strategies aimed at improving diet quality in Kazakhstan should consider these factors and address the challenges posed by dietary transition, with an emphasis on preserving beneficial traditional eating habits while reducing the impact of energy-dense, processed foods.

## Data Availability

The raw data supporting the conclusions of this article will be made available by the authors, without undue reservation.

## References

[1] Gherasim A Arhire LI Nita O Popa AD Graur M Mihalache L. The relationship between lifestyle components and dietary patterns. Proc Nutr Soc. (2020) 79:311–23. doi: 10.1017/S0029665120006898, PMID: 32234085 PMC7663317

[2] Azimi MN Rahman MM Maraseni T. The interplay of dietary habits, economic factors, and globalization: assessing the role of institutional quality. Nutrients. (2024) 16:3116. doi: 10.3390/nu16183116, PMID: 39339718 PMC11435435

[3] Popkin BM Ng SW. The nutrition transition to a stage of high obesity and noncommunicable disease prevalence dominated by ultra-processed foods is not inevitable. Obes Rev. (2022) 23:e13366. doi: 10.1111/obr.13366, PMID: 34632692 PMC8639733

[4] Neuhouser ML. The importance of healthy dietary patterns in chronic disease prevention. Nutr Res. (2019) 70:3–6. doi: 10.1016/j.nutres.2018.06.002, PMID: 30077352 PMC6328339

[5] Rahimon RM. Evolution of land use in pastoral culture in Central Asia with special reference to Kyrgyzstan and Kazakhstan In: Squires V, editor. Rangeland stewardship in Central Asia. Dordrecht: Springer Netherlands (2012). 51–67.

[6] Lightfoot E Motuzaite-Matuzeviciute G O'Connell TC Kukushkin IA Loman V Varfolomeev V. How ‘pastoral’ is pastoralism? Dietary diversity inBronzeAgeCommunities in the CentralKazakhstan steppes. Archaeometry. (2014) 57:232–49. doi: 10.1111/arcm.12123

[7] Jia M Zhen L Xiao Y. Changing food consumption and nutrition intake in Kazakhstan. Nutrients. (2022) 14:326. doi: 10.3390/nu14020326, PMID: 35057506 PMC8778289

[8] Fanzo J McLaren R Bellows A Carducci B. Challenges and opportunities for increasing the effectiveness of food reformulation and fortification to improve dietary and nutrition outcomes. Food Policy. (2023) 119:102515. doi: 10.1016/j.foodpol.2023.102515

[9] Sandri E Cantin Larumbe E Capoferri M Cerda Olmedo G Werner LU Vega-Bello MJ. Socio-demographic determinants of dietary choices and their impact on health in Spanish adults. Front Public Health. (2024) 12:1417925. doi: 10.3389/fpubh.2024.1417925, PMID: 39575104 PMC11578831

[10] Marchioni DM Latorre MR Eluf-Neto J Wunsch-Filho V Fisberg RM. Identification of dietary patterns using factor analysis in an epidemiological study in São Paulo. Sao Paulo Med J. (2005) 123:124–7. doi: 10.1590/s1516-31802005000300007, PMID: 16021275 PMC11060379

[11] Newby PK Tucker KL. Empirically derived eating patterns using factor or cluster analysis: a review. Nutr Rev. (2004) 62:177–203. doi: 10.1301/nr.2004.may.177-203, PMID: 15212319

[12] Thorpe MG Milte CM Crawford D McNaughton SA. Education and lifestyle predict change in dietary patterns and diet quality of adults 55 years and over. Nutr J. (2019) 18:67. doi: 10.1186/s12937-019-0495-6, PMID: 31699092 PMC6839215

[13] Hair Junior JF Black WC Babin BJ Anderson RE Tatham RL. Multivariate data analysis. Upper Saddle River, New Jersey, USA. (1998).

[14] Barros AJ Hirakata VN. Alternatives for logistic regression in cross-sectional studies: an empirical comparison of models that directly estimate the prevalence ratio. BMC Med Res Methodol. (2003) 3:1–13. doi: 10.1186/1471-2288-3-21 14567763 PMC521200

[15] Hu FB. Diet strategies for promoting healthy aging and longevity: an epidemiological perspective. J Intern Med. (2024) 295:508–31. doi: 10.1111/joim.13728, PMID: 37867396 PMC10939982

[16] Sousa S Lanca de Morais I Albuquerque G Gelormini M Casal S Pinho O. Patterns of street food purchase in cities from Central Asia. Front Nutr. (2022) 9:925771. doi: 10.3389/fnut.2022.925771, PMID: 35811986 PMC9263728

[17] Feraco A Armani A Amoah I Guseva E Camajani E Gorini S. Assessing gender differences in food preferences and physical activity: a population-based survey. Front Nutr. (2024) 11:1348456. doi: 10.3389/fnut.2024.1348456, PMID: 38445208 PMC10912473

[18] Warren A. The relationship between gender differences in dietary habits, neuroinflammation, and Alzheimer's disease. Front Aging Neurosci. (2024) 16:1395825. doi: 10.3389/fnagi.2024.1395825, PMID: 38694261 PMC11061392

[19] Horlyck-Romanovsky MF Huang TT Ahmed R Echeverria SE Wyka K Leung MM. Intergenerational differences in dietary acculturation among Ghanaian immigrants living in new York City: a qualitative study. J Nutr Sci. (2021) 10:e80. doi: 10.1017/jns.2021.69, PMID: 34616551 PMC8477345

[20] Mills S Brown H Wrieden W White M Adams J. Frequency of eating home cooked meals and potential benefits for diet and health: cross-sectional analysis of a population-based cohort study. Int J Behav Nutr Phys Act. (2017) 14:109. doi: 10.1186/s12966-017-0567-y, PMID: 28818089 PMC5561571

[21] Merchant AT. Grand challenges in oral health and nutrition: we are what we eat. Front Oral Health. (2022) 3:999817. doi: 10.3389/froh.2022.999817, PMID: 36092139 PMC9448949

[22] Castro-Barquero S Ruiz-Leon AM Sierra-Perez M Estruch R Casas R. Dietary strategies for metabolic syndrome: a comprehensive review. Nutrients. (2020) 12:2983. doi: 10.3390/nu12102983, PMID: 33003472 PMC7600579

[23] Hu FB. Are refined carbohydrates worse than saturated fat? Am J Clin Nutr. (2010) 91:1541–2. doi: 10.3945/ajcn.2010.29622, PMID: 20410095 PMC2869506

[24] Bahadoran Z Mirmiran P Azizi F. Fast food pattern and cardiometabolic disorders: a review of current studies. Health Promot Perspect. (2015) 5:231–40. doi: 10.15171/hpp.2015.028, PMID: 26933642 PMC4772793

[25] Maksimov S Karamnova N Shalnova S Drapkina O. Sociodemographic and regional determinants of dietary patterns in Russia. Int J Environ Res Public Health. (2020) 17:328. doi: 10.3390/ijerph17010328, PMID: 31947733 PMC6981481

[26] Bromage S Daria T Lander RL Tsolmon S Houghton LA Tserennadmid E. Diet and nutrition status of Mongolian adults. Nutrients. (2020) 12:1514. doi: 10.3390/nu12051514, PMID: 32456038 PMC7284332

[27] Mengmeng J Lin Z Changshun Z. Analysis of food consumption and its characteristics in Uzbekistan based on the emergy method. J Resour Ecol. (2022) 13:842–50. doi: 10.5814/j.issn.1674-764x.2022.05.008

[28] Aghayan M Asghari G Yuzbashian E Mahdavi M Mirmiran P Azizi F. Secular trend in dietary patterns of Iranian adults from 2006 to 2017: Tehran lipid and glucose study. Nutr J. (2020) 19:110. doi: 10.1186/s12937-020-00624-x, PMID: 33010805 PMC7533031

