# Supplementary material for: Socioeconomic and demographic factors influencing dietary patterns in Aktobe: a sample of Kazakhstan’s dietary culture
Source: Front Public Health. 2025 Oct 29;13:1687222. doi: 10.3389/fpubh.2025.1687222 (PMC12605201; doi:10.3389/fpubh.2025.1687222)
Supplement: Supplementary file 1 [file Data_Sheet_1.docx]

# **Appendix A. Variable definitions and coding**

Anthropometric and clinical variables

- Body mass index (BMI): Calculated as weight (kg) / height² (m²).
  - *Height and weight* were measured using a calibrated stadiometer and scale.
  - BMI reported as continuous variable.
- Blood pressure: Systolic and diastolic pressures were measured twice after a 5-minute rest in a seated position using an automated sphygmomanometer; the mean value was used.

Sociodemographic variables

- Age: Recorded in years; categorized into four groups:
  - 18-24 years (code 1)
  - 25-39 years (code 2)
  - 40-59 years (code 3)
  - 60-65 years (code 4)
- Gender: Male = 1; Female = 0.
- Labor status: Works (1); Does not work (2); Pensioner (3); Student (4).
- Family size: Number of household members, coded as 1, 2, 3, 4, 5, 6, 7.
- Monthly family income: Collected in USD (continuous).
- Nutritional expenses: Collected in KZT (continuous).
- Proportion of income spent on food: Expressed as % of total monthly income.

Lifestyle variables

- Number of meals per day: 3, 4, or 5.
- Minimum interval between meals (hours): 2, 3, or 4.
- Maximum interval between meals (hours): 4, 5, 6, 7, or 8.
- Days per week without breakfast at work: 0-5 days.
- Dry food consumption: Yes = 1; No = 0.
- Eating just before bedtime: Yes = 1; No = 0.
- Most satisfying meal: Lunch (1) or Dinner (2).
- Smoking (number of cigarettes per day):
  - 0 = non-smoker
  - 1–9, 10–15, >15 (reference).
- Alcohol consumption: Self-reported, recorded as mL/week (continuous).

Health status variables

- Self-rated health: Excellent = 1; Good = 2.
- Doctor visit in past 3 months: Yes = 1; No = 0.
- Oral health indicators:
  - Bleeding gums when brushing: No (0), Sometimes (1), Often (2).
  - Large number of carious teeth: No (0), Sometimes (1), Often (2).
- Reported symptoms: Nausea, loss of appetite, cracks in mouth corners, shortness of breath during exercise, irritability/anxiety/sleep disturbance/memory or attention loss, intolerance to bright light, decreased vision at dusk, dry skin/brittle nails, fatigue/weakness/pain in calf muscles, pain in right hypochondrium, epigastric pain, weakening of physical performance, mental disability, frequency of colds (no more than once/year vs. ≥2 times).
  - All coded as: No (0), Sometimes (1), Often (2).
- Healthcare behavior:
  - Consults a doctor if unwell: Yes = 1; No = 0.
  - Always takes sick leave if ill: Yes = 1; No = 0.

Chronic disease variables

- Gastrointestinal diseases: Yes = 1; No = 0.
- Cardiovascular diseases: Yes = 1; No = 0 (includes hypertension, ischemic heart disease, arrhythmia).
- Endocrine diseases: Yes = 1; No = 0 (includes thyroid disease, diabetes mellitus).
- Kidney diseases: Yes = 1; No = 0.
- Diabetes mellitus: Yes = 1; No = 0.
- Liver diseases: Yes = 1; No = 0.
- Other diseases: Yes = 1; No = 0.

Questionnaires

- Dietary intake: Collected using a structured food frequency questionnaire (FFQ) adapted for the Kazakh population. Foods were grouped into 11 categories (beverages; grains; legumes; meat; dairy; eggs; vegetables; fruits; sweets/desserts; condiments/sauces; sugar).
- Self-rated health and oral health were assessed using simple structured questions embedded in the same survey.

# **Appendix B. Technical details of z-standardization and score calculation**

The standardized value of an item (food group) in this context referred to the z-score of the item. The z-score measured how many standard deviations an individual data point was from the mean of the dataset. Here's how the standardized value for each food group was calculated:

Calculation of the Mean: The mean (average) value of the food group across all individuals was determined.

Calculation of the Standard Deviation: The standard deviation of the food group across all individuals was determined.

Calculation of the Z-Score: For each individual's food group value, the mean of the food group was subtracted and divided by the standard deviation.

The formula for the z-score (standardized value) is: Z=(X-μ)/σ; where Z is the standardized value (z-score), X is the individual's food group value, μ is the mean of the food group values, and σ is the standard deviation of the food group values.

For each retained factor, the individual's score was calculated by summing the standardized values of the items (food groups), each weighted by its absolute scoring coefficient (factor loading). The formula for the score was: Score = ∑(Zi×∣Li∣); where Zi is the standardized value of the i-th food group and Li is the factor loading (scoring coefficient) of the i-th food group.

Each individual received a score for each retained factor, calculated by summing the standardized values of the items (food groups) weighted by their absolute scoring coefficients (factor loadings). The dietary patterns were categorized into quartiles, with the upper quartile representing the highest adherence to the pattern.

# **Appendix C.** **Detailed results of bivariate regression analyses of predictors of dietary pattern adherence**

Table C1. Bivariate regression analyses of sociodemographic predictors of dietary pattern adherence (negative binomial regression, n=460)

| Predictor (reference group) | B (Coef.) | 95% CI (Lower-Upper) | Wald χ² | p-value |
| --- | --- | --- | --- | --- |
| Sex (female = ref) | -0.12 | -0.30 to 0.06 | 2.12 | 0.15 |
| Age 18-24 (vs. 60-65) | -0.39 | -0.70 to -0.08 | 5.44 | 0.02 |
| Age 25-39 (vs. 60-65) | -0.28 | -0.55 to -0.01 | 4.15 | 0.04 |
| Age 40-59 (vs. 60-65) | -0.37 | -0.65 to -0.10 | 7.35 | 0.007 |
| Labor status: works (vs. student) | 0.24 | -0.17 to 0.65 | 1.31 | 0.25 |
| Labor status: doesn’t work | 0.20 | -0.39 to 0.78 | 0.43 | 0.51 |
| Labor status: pensioner | 0.28 | -0.32 to 0.88 | 0.83 | 0.36 |
| Family size 1 (vs. ≥7) | -0.40 | -1.05 to 0.25 | 1.49 | 0.22 |
| Family size 2 | -0.23 | -0.89 to 0.42 | 0.48 | 0.49 |
| Family size 3 | 0.06 | -0.54 to 0.67 | 0.05 | 0.83 |
| Family size 4 | -0.35 | -0.96 to 0.26 | 0.41 | 0.74 |
| Family size 5 | -0.11 | -0.74 to 0.53 | 0.11 | 0.74 |
| Family size 6 | -0.11 | -0.76 to 0.54 | 0.12 | 0.74 |

Table C2. Bivariate regression analyses of lifestyle predictors of dietary pattern adherence (negative binomial regression, n=460)

| Predictor (reference group) | B (Coef.) | 95% CI (Lower-Upper) | Wald χ² | p-value |
| --- | --- | --- | --- | --- |
| Meals/day = 3 (vs. 5) | 0.11 | -0.36 to 0.57 | 0.19 | 0.66 |
| Meals/day = 4 (vs. 5) | 0.59 | 0.05 to 1.12 | 4.67 | 0.03 |
| Min. interval 2h (vs. 4h) | 0.54 | 0.08 to 1.00 | 5.42 | 0.02 |
| Min. interval 3h (vs. 4h) | 0.55 | 0.31 to 0.79 | 20.0 | <0.001 |
| Max. interval 5h (vs. 8h) | 0.54 | -1.08 to 2.16 | 0.44 | 0.51 |
| Max. interval 6h (vs. 8h) | 0.94 | -0.67 to 2.54 | 1.33 | 0.25 |
| Max. interval 7h (vs. 8h) | 1.01 | -0.60 to 2.62 | 1.52 | 0.22 |
| Without breakfast, 0 days (vs. 5) | 0.41 | 0.12 to 0.71 | 7.25 | 0.007 |
| Without breakfast, 1 day | 0.14 | -0.24 to 0.52 | 0.53 | 0.47 |
| Without breakfast, 2 days | 0.19 | -0.13 to 0.51 | 1.32 | 0.25 |
| Without breakfast, 3 days | 0.18 | -0.20 to 0.55 | 0.84 | 0.36 |
| Without breakfast, 4 days | 0.30 | -0.08 to 0.68 | 2.46 | 0.12 |
| Dry food (No vs. Yes) | 0.29 | 0.06 to 0.53 | 5.28 | 0.02 |
| Eating just before bedtime (No) | -0.07 | -0.26 to 0.13 | 0.45 | 0.50 |
| Most satisfying meal (Lunch vs. Dinner) | 0.03 | -0.18 to 0.23 | 0.06 | 0.81 |
| Smoking 0/day (ref ≥15/day) | 0.01 | -0.45 to 0.48 | 0.00 | 0.95 |
| Smoking 5/day | -1.21 | -2.12 to -0.30 | 6.74 | 0.009 |
| Smoking 6/day | 0.15 | -0.43 to 0.72 | 0.24 | 0.62 |
| Smoking 7/day | 0.60 | -0.08 to 1.28 | 3.04 | 0.08 |
| Smoking 8/day | -0.09 | -0.85 to 0.66 | 0.06 | 0.81 |
| Smoking 10/day | 0.08 | -0.50 to 0.67 | 0.08 | 0.78 |

Table C3. Bivariate regression analyses of health-related predictors of dietary pattern adherence (negative binomial regression, n=460)

| Predictor (reference group) | B (Coef.) | 95% CI (Lower-Upper) | Wald χ² | p-value |
| --- | --- | --- | --- | --- |
| Oral health |  |  |  |  |
| Bleeding gums (No vs. Often) | 0.42 | 0.01 to 0.84 | 3.91 | 0.05 |
| Carious teeth (No vs. Often) | 0.72 | 0.24 to 1.21 | 8.93 | 0.003 |
| Carious teeth (Sometimes vs. Often) | 0.46 | -0.04 to 0.95 | 3.36 | 0.07 |
| Symptoms and functional status |  |  |  |  |
| Shortness of breath (No vs. Often) | 0.33 | 0.03 to 0.63 | 4.62 | 0.03 |
| Shortness of breath (Sometimes vs. Often) | 0.23 | -0.07 to 0.53 | 2.23 | 0.13 |
| Weakening of physical performance (No vs. Often) | 0.99 | 0.59 to 1.39 | 13.6 | <0.001 |
| Weakening of physical performance (Sometimes vs. Often) | 0.73 | 0.38 to 1.09 | 13.1 | <0.001 |
| Mental disability (No vs. Often) | 1.04 | 0.37 to 1.71 | 9.69 | 0.002 |
| Mental disability (Sometimes vs. Often) | 0.79 | 0.12 to 1.46 | 5.24 | 0.022 |
| Fatigue/weakness (No vs. Often) | -0.05 | -0.44 to 0.34 | 0.06 | 0.80 |
| Fatigue/weakness (Sometimes vs. Often) | 0.05 | -0.39 to 0.49 | 0.05 | 0.82 |
| Dry skin, brittle nails (No vs. Often) | -0.01 | -0.36 to 0.34 | 0.00 | 0.96 |
| Dry skin, brittle nails (Sometimes vs. Often) | 0.11 | -0.34 to 0.56 | 0.25 | 0.62 |
| Epigastric pain (No vs. Often) | -0.01 | -0.47 to 0.44 | 0.00 | 0.96 |
| Epigastric pain (Sometimes vs. Often) | -0.01 | -0.57 to 0.55 | 0.00 | 0.98 |
| Nausea (No vs. Often) | 0.10 | -0.28 to 0.48 | 0.26 | 0.61 |
| Loss of appetite (No vs. Often) | -0.22 | -0.50 to 0.06 | 2.38 | 0.12 |
| Irritability/sleep disturbance (No vs. Often) | 0.01 | -0.30 to 0.32 | 0.01 | 0.93 |
| Chronic diseases |  |  |  |  |
| Endocrine disease (No vs. Yes) | 0.55 | 0.21 to 0.89 | 9.65 | 0.002 |
| Cardiovascular disease (No vs. Yes) | -0.08 | -0.34 to 0.18 | 0.36 | 0.55 |
| Gastrointestinal disease (No vs. Yes) | -0.15 | -0.35 to 0.04 | 2.27 | 0.13 |
| Liver disease (No vs. Yes) | -0.37 | -1.03 to 0.29 | 1.20 | 0.27 |
| Diabetes mellitus (No vs. Yes) | 0.24 | -0.35 to 0.84 | 0.64 | 0.42 |
| Kidney disease (No vs. Yes) | ND | ND | ND | ND |
| Other chronic disease (No vs. Yes) | 0.03 | -0.26 to 0.32 | 0.04 | 0.83 |
